# Evaluating the Consistency of Gene Methylation in Liver Cancer Using Bisulfite Sequencing Data

**DOI:** 10.3389/fcell.2021.671302

**Published:** 2021-04-29

**Authors:** Xubin Zheng, Qiong Wu, Haonan Wu, Kwong-Sak Leung, Man-Hon Wong, Xueyan Liu, Lixin Cheng

**Affiliations:** ^1^Department of Critical Medicine, Shenzhen People’s Hospital, First Affiliated Hospital of Southern University of Science and Technology, Second Clinical Medicine College of Jinan University, Shenzhen, China; ^2^Department of Computer Science and Engineering, The Chinese University of Hong Kong, Hong Kong, China; ^3^School of Biomedical Sciences, The Chinese University of Hong Kong, Hong Kong, China

**Keywords:** whole-genome bisulfite sequencing, reduced-representation bisulfite sequencing, targeted bisulfite sequencing, liver cancer, DNA methylation

## Abstract

Bisulfite sequencing is considered as the gold standard approach for measuring DNA methylation, which acts as a pivotal part in regulating a variety of biological processes without changes in DNA sequences. In this study, we introduced the most prevalent methods for processing bisulfite sequencing data and evaluated the consistency of the data acquired from different measurements in liver cancer. Firstly, we introduced three commonly used bisulfite sequencing assays, i.e., reduced-representation bisulfite sequencing (RRBS), whole-genome bisulfite sequencing (WGBS), and targeted bisulfite sequencing (targeted BS). Next, we discussed the principles and compared different methods for alignment, quality assessment, methylation level scoring, and differentially methylated region identification. After that, we screened differential methylated genes in liver cancer through the three bisulfite sequencing assays and evaluated the consistency of their results. Ultimately, we compared bisulfite sequencing to 450 k beadchip and assessed the statistical similarity and functional association of differentially methylated genes (DMGs) among the four assays. Our results demonstrated that the DMGs measured by WGBS, RRBS, targeted BS and 450 k beadchip are consistently hypo-methylated in liver cancer with high functional similarity.

## Introduction

Epigenetics investigates the heritable changes of gene activity or function not caused by DNA sequences, such as mutations, deletions, insertions, and translocation. One of the major mechanisms in epigenetics is DNA methylation, which is a chemical transformation that happened in the DNA strand. DNA methylation, accounting for around 1.5% of human genomic DNA, usually refers to the addition of the methyl group to the fifth carbon of cytosine (C), forming 5-methylcytosine (5 mC). In human beings, DNA methylation mainly occurs at the site of a cytosine followed by a guanidine nucleotide, which is called the CpG site, but it may also happen in non-CpG contexts (Moore et al., 2013). CpG sites were revealed to be nonuniformly distributed and tend to cluster together. CpG island is defined as a cluster of CpG sites where the fraction of CG interstrand base pair is greater than 0.5 and the CpG ratio is greater than 0.6 within more than 200-bp regions. If methylation happens on CpG island in the promoter, the gene expression is repressed. Besides CpG site, DNA methylation is less-frequently found in non-CpG contexts (e.g., CHG and CHH, where H = A, T or C).

DNA methylation is of vital importance in numerous developmental, physiologic and pathologic processes (Moore et al., 2013). DNA methylation pattern is distinct between cell types, developmental states and different disease situations. Faults in DNA methylation may result in lesion in liver cancer, which is the third most fatal and the sixth commonly diagnosed cancer in 2020 with more than 905,600 new cases and 830,000 new deaths (Sung et al., 2021). Understanding the epigenetic phenomenon of liver cancer may contribute to the early diagnosis, which is vital for curing liver cancer *via* partial hepatectomy or liver transplantation (Villanueva et al., 2013). Aberrant p16 methylation was detected in both hepatocellular carcinoma tissues and plasma/serum samples by Wong et al. (1999) two decades ago. Yao et al. (2000) found that the methylation status of γ-glutamyl transferase gene altered and γ-glutamyl transferase expressed abnormally in hepatocellular carcinoma. Stefanska et al. (2011) identified around 3,700 hypomethylated promotors in liver cancer samples.

Many methods have been developed to measure DNA methylation including affinity enrichment and bisulfite-based ones. Bisulfite sequencing methods are usually considered as the golden standard, owing to its high resolution, flexibility across organisms, and low input requirements (Yong et al., 2016). By using sodium bisulfite treatment, the epigenetic information can be transformed into genetic information and therefore can be assessed by sequencing methods. The principle of bisulfite sequencing is a chemical reaction of bisulfite conversion that transforms the unmethylated cytosine residues to uracil residues, while the methylated cytosine remains unchanged. The DNA is fragmented after bisulfite conversion due to the chemical treatment. PCR amplification will then be applied and uracils will be replaced by thymines. When sequencing is performed, the methylated cytosines become thymines. Compared with the reference-unconverted sequence, the methylated cytosines can be distinguished.

Bisulfite sequencing methods include whole-genome bisulfite sequencing (WGBS), reduced representation bisulfite sequencing (RRBS), targeted bisulfite sequencing (targeted-BS), etc. In this study, we mainly focus on the computational data processing for the popular methods WGBS, RRBS and targeted-BS in liver cancer. WGBS sequences the whole genome and therefore covers all the cytosine information in theory (Lister et al., 2009). The genomic DNA is purified from tissue and cut into fragments. Bisulfite treatment is then performed on DNA fragments to convert unmethylated cytosine (C) into uracil (U). Bisulfite converted DNA fragments are primed randomly by polymerase to synthesize sequence tags. Resulting strands are selected to synthesize with another sequence tag at 3′ end and become di-tagged DNA with a known sequence at both ends. The tags can be combined with adapters for PCR amplification. After PCR amplification, the bisulfite-converted strand will be sequenced. WGBS has several advantages, such as high coverage of nearly every CpG site, detection of partially methylated domains, and acquirement of absolute DNA methylation level. Moreover, it can detect methylation in the non-CG context. However, WGBS is expensive and labor-intensive due to the process of the whole genome.

To measure methylome at a lower cost, RRBS (Meissner et al., 2005) was proposed to investigate the regions with high methylation probability. DNA is digested by Msp1 restriction enzyme, which cuts at CCGG sites. It improves the CpG enrichment in the fragments and covers 85% of the CpG islands, mostly in promoters. The fragment ends are then ligated by adapters and selected with sizes between 40 and 220 bps. Next, bisulfite treatment, PCR amplification and sequencing were applied. The digested and selected fragments only compose 1–3% of the genome and hence save the cost of sequencing. RRBS is more efficient than WGBS because it focuses on the CpG rich regions, but it loses information due to the lack of coverage at some less studied areas and cannot cover most of the non-CG methylation.

Targeted BS was developed to measure the methylome of more specific regions, such as exome and gene promotors. It may require a hybridization step to capture targeted methylated regions with pre-designed oligos. Targeted BS can obtain single-base resolution DNA methylation patterns and thus achieve enhanced accuracy and sensitivity with efficient cost. However, the oligos need to be designed for different targets. One of the targeted BS techniques developed in liver cancer is the liquid hybridization capture-based bisulfite sequencing (LHC-BS), which applied biotinylated RNA probes to capture target regions.

Other than bisulfite sequencing, microarrays such as the Infinium© HumanMethylation450 BeadChip (450 k) are widely used in methylation measurement for its high throughput and low cost. The 450 k covers 480,000 CpG sites *via* target-specific probes. Two types of probes are applied to CpG locus, one for methylated cytosine or converted thymine and the other for the complements of upstream. Methylation levels are obtained by comparing the two probe intensities.

In this study, we first introduced the procedures of processing bisulfite sequencing data, including alignment, quality control, methylation level scoring, and differentially methylated region identification. Then we compared the popular tools from different aspects. Next, we screened differential methylated genes in liver cancer through three bisulfite sequencing and compared their consistency. Lastly, we made a comparison of the results from bisulfite sequencing to 450 k microarray.

## Materials and Methods

A general bisulfite sequencing data process consists of adapter trimming, alignment, quality control, methylation level scoring, and differential methylation region identification. We details all the steps except the trimming step, which is simple and the normalization step is the same as DNA sequencing.

### Aligning Bisulfite Sequencing Reads

Different from the alignment in DNA sequencing, aligning the bisulfite sequencing reads to the reference genome is challenging because the unmethylated cytosines (C) are converted to thymines (T) after bisulfite treatment. That means most of the T in the bisulfite sequence should be mapped to C in the reference genome.

Mostly two types of approaches, three-letter alignment and wildcard alignment, are applied. The most popular three-letter aligner in the past decade was Bismark (Krueger and Andrews, 2011). In Bismark, all the Cs in bisulfite reads and reference genome was converted into Ts to perform alignment and thereby only three letters, A, T and G were left. When C was converted to T after bisulfite treatment, in the opposite strand the G becomes A with PCR amplification. Therefore, in Bismark the alignment was run once again with all the G converted to A in both bisulfite sequence and reference sequence. As the sequence was converted into three-letter alignment, Bismark applied Bowtie (Langmead et al., 2009), which is a famous aligner for DNA sequence, to map the reads into a reference genome. In the end, a comparison between different strands was made to determine which part of the reference genome to map. Moreover, the methylated loci were pointed out by comparing the bisulfite sequence and the reference gene. Bismark toolbox keeps update till now and is available for WGBS, RRBS and targeted BS data. Although it may introduce error when converting C to T and G to A, it worked fast as compared to the BSMAP (Xi and Li, 2009), a wildcard aligner.

BSMAP (Xi and Li, 2009) was a representative wildcard aligner. Different from the three-letter aligner, wildcard aligner replaced Cs with Ys in the reference genome and allowed both Cs and Ts in bisulfite reads to align to Ys. For each part of the sequence in the reference genome, BSMAP built a seed table that listed all the possible reads. Then part of the reads was mapped to the potential references as key and checked whether the rest of the reads were matched. It was more accurate than Bismark because it enumerated all the possibilities, but on the other hand less efficient.

Most aligners proposed in recent years applied three-letter alignment due to its ability for large data size. BRAT-nova (Harris et al., 2016) applied hash table and concatenated two strands together to align to the reference genome instead of aligning two times, resulting in high efficiency than Bismark. It also supported single variable-length indel caused by mutation and hence had better map ability. BatMeth2 (Zhou et al., 2019) focused more on the indel during mapping. It allowed five variable length mismatch and achieved high accuracy and map ability. VAliBS (Li et al., 2017) discovered that some unmapped reads were due to introducing primer during the assay. Hence after aligning using Bismark, it trimmed the unmapped reads and ran alignment again so that more reads can be mapped to reference. Moreover, it provided a graphical user interface for non-programmers. Since alignment is computationally heavy, a natural way to improve efficiency is to compute in parallel. BiSpark (Soe et al., 2018) used Spark engine to execute the three-letter alignment parallelly on the distributed system with load balance. It only took 1/3 to half the time of Bismark according to their results. BS Seeker3 (Huang et al., 2018) combined the hash table and three-letter aligner and allowed longer reads to be aligned. Better accuracy was achieved by BS Seeker3. The features of the aligner mentioned were compared in Table 1.

**TABLE 1:** Aligners for bisulfite sequencing data.

Methods	Years	Types	Features	GUI
BatMeth2 (Zhou et al., 2019)	2019	Three-letter aligner	indel sensitive	No
BS Seeker3 (Huang et al., 2018)	2018	Three-letter aligner	hase table with greater length	No
BiSpark (Soe et al., 2018)	2018	Three-letter aligner	distributed system, load-balanced	No
VAliBS (Li et al., 2017)	2017	Three-letter aligner	improve accuracy by trimming unmapped read	Yes
BRAT-nova (Harris et al., 2016)	2016	Three-letter aligner	hash table with concatenate two strands, supports a single variable-length indel	No
Bismark (Krueger and Andrews, 2011)	2011	Three-letter aligner	methylated visualization in command line	No
BSMAP (Xi and Li, 2009)	2009	Wildcard	hase table, mismatch counting	No

### Quality Control

Quality control is applied to evaluate the assay quality and aligning quality aiming at finding out whether the results are trustworthy. Typical metrics include the number of mapped and unmapped reads, the read coverage at CpG sites, and the bisulfite conversion rate.

A (fire)cloud-based platform proposed in 2019 by Kangeyan et al. (2019) involved a lot of metrics for quality assessment including Read metrics, CpG Coverage, M-bias, Downsampling saturation curve, CpG discretization, Feature level coverage, Bisulfite conversion rate, CpG density distribution, and Methylation distribution. The pipeline RnBeads 2.0 (Muller et al., 2019) provided quality control, but only focused on the read coverage of each site. It also provided visualization of the quality results. BS Seeker3 (Huang et al., 2018) calculated the average rate of mismatch per read position for quality assessment. MethGo (Liao et al., 2015) provided visualization of metrics from different aspects including coverage distribution and methylation level distribution. GBSA (Benoukraf et al., 2013) assessed the quality with the depth of coverage for each cytosine site of interest, and the ratio of sequenced cytosine to the total amount of cytosine within the domain. It provided a graphical interface, which is more user-friendly. The features of quality control methods were summarized in Table 2.

**TABLE 2:** Quality control methods for bisulfite sequencing data.

Methods	Year	Features	GUI
(Fire)cloud-based platform (Kangeyan et al., 2019)	2019	Read metrics, CpG Coverage…, Fast	No
RnBeads2 (Muller et al., 2019)	2019	based on read coverage, visualization	Yes
BS Seeker3 (Huang et al., 2018)	2018	average rate of mismatch per read position	No
MethGo (Liao et al., 2015)	2015	coverage distribution of methylation sites, other metrics for analysis, visualization	No
GBSA (Benoukraf et al., 2013)	2013	depth of coverage for each cytosine site of interest, the ratio of sequenced cytosine to the total amount of cytosine within the domain	Yes

*GUI: Graph user interface.*

### Methylation Level Scoring

If the quality of the alignment results is acceptable, the methylation level is calculated for each methylation site. The major principle is to calculate the fraction of methylated reads that cover the sites. The basic formula is

$$\mathrm{Methylation}\mathrm{Level}=\frac{C}{\left(C+T\right)}\times 100\%$$

where C and T represent the number of cytosines and thymines among all reads in the site.

BatMeth2 (Zhou et al., 2019) divided the situations into high coverage which took SNP into consideration, and low coverage which used the original formula. It improved the accuracy for the high coverage situation. BS Seeker 3 (Huang et al., 2018) provided a visualization of the methylated level in the whole genome. GBSA (Benoukraf et al., 2013) offered a graphical interface for methylation level scoring. Besides the methylation level, the BSPAT (Hu et al., 2015) used Z-score to evaluate the significance based on read coverage. It output graphs to show the methylation levels and significance for the genome. Moreover, it is an online tool so that users can run it for a large quantity of data. The above methods were compared in Table 3.

**TABLE 3:** Methylation level scoring tools for bisulfite sequencing.

Methods	Year	Features	GUI
BatMeth2 (Zhou et al., 2019)	2019	divide high coverage and low coverage	No
BS Seeker3 (Huang et al., 2018)	2018	genome-wide view of methylation levels	No
BSPAT (Hu et al., 2015)	2015	3 types of visualization, Z-score for significance, online	Yes
GBSA (Benoukraf et al., 2013)	2013	results visualization	Yes

### Differentially Methylated Region Identification

To reveal the methylation patterns in different stages, differentially methylated region (DMR) identification/calling is performed by comparing the methylation levels between control and case samples with statistical methods. Classical hypothesis testing methods can be applied for DMR calling such as fisher’s exact test, chi-square test, *t*-test, Goeman’s global test and analysis of variance (ANOVA). These methods can be categorized into count-based hypothesis tests and ratio-based hypothesis tests.

Count-based hypothesis tests regard the methylation level within regions on each sample as categorical variable. By counting the number of methylated and unmethylated samples of control and case groups, a contingency table is built. Fisher’s exact test, which calculates whether the region is significantly differential, is the most commonly used. Besides Fisher’s exact test, Chi-square method can be applied to select differential region, but also for multiple groups. Logistic regression approaches assume the read counts follow a Poisson distribution and apply the Wald test to evaluate the difference between two Poisson means. Ratio-based hypothesis tests compare the methylation rate between groups by taking the ratio of methylated read counts and total read counts. *T*-test and moderate *t*-test are used for two classes, and ANOVA can be applied for multi-group comparison.

Most of the tools provide both count-based and ratio-based methods for different read coverage. A well-known tool kit methlKit (Akalin et al., 2012) provided logistic regression and Fisher’s exact test for users to choose. DMAP (Stockwell et al., 2014) implemented ANOVA, chi-square test for multiple groups other than Fisher’s exact test. RnBead2 (Muller et al., 2019) applied Fisher’s method but ranked the differential regions by adjusting p-value, difference in variance, and size effect. All these methods applied the false discovery rate (FDR) correction to adjust P-value for multiple tests.

Other than the classical hypothesis test, the hidden Markov model, which models the methylation level of the CpG sites as methylation states, was once applied such as ComMet (Li et al., 2013). DMRFusion (Yassi et al., 2018) integrated Information gain, Between versus within Class scatter ratio, Fisher ratio, Z-score, and Welch’s *t*-test by converting into rank and combining together. HOME (Srivastava et al., 2019) built a histogram of methylation reads region by region and selected DMR by support vector machine. MethCP (Gong and Purdom, 2020) was one of the latest papers for DMR. It included the spatial information of regions by circular binary segmentation and applied Fisher’s combined probability test for p-value. It took into account the weighted-sum effect size and variation for time-course study. These methods were compared in Table 4.

**TABLE 4:** DMR methods for bisulfite sequencing.

Methods	Modeling	Features
MethCP (Gong and Purdom, 2020)	Circular Binary Segmentation (segment with significantly different mean), Fisher’s combined probability test	weight sum effect size and variation, time course
RnBead2 (Muller et al., 2019)	Combined ranking of 1. absolute difference in mean DNA methylation levels 2. relative difference 3. p-value (Fisher’s method)	GUI, parallelization and automatic distribution
HOME (Srivastava et al., 2019)	Histogram, Support Vector Machine	Learning methods for prediction
DMRFusion (Yassi et al., 2018)	Information gain, Between versus within Class scatter ratio, Fisher ratio, Z-score and Welch’s *t*-test	rank the metrics and combine together
DMAP (Stockwell et al., 2014)	Fisher’s Exact test, ANOVA, chi-square test	
ComMet (Li et al., 2013)	Hidden Markov model	
methlKit (Akalin et al., 2012)	logistic regression, Fisher’s Exact Test	

### Analysis of WGBS, RRBS, and Targeted BS Datasets

We collected three types of publicly available data sets (Table 5) from Gene Expression Omnibus (GEO) (Edgar et al., 2002) to analyze the consistency of WGBS, RRBS and targeted BS in liver cancer. Only one WGBS data set [GSE70090 (Li et al., 2016)], one RRBS data set [GSE112221 (Hlady et al., 2019)] and one targeted BS data set [GSE55752 (Gao et al., 2015)] measuring liver cancer tissue were found using keywords “liver cancer” / “Hepatocellular Carcinoma” and “bisulfite sequencing.” The GSE70090 data set detected three liver cancer samples and three normal controls using WGBS. The GSE112221 data set includes four hepatocellular carcinomas (HCC) and six controls containing four cirrhosis and two normals measured by RRBS. The GSE55752 captured methylation from eight pairs of HCC tumor and non-tumor liver samples using a type of targeted BS approach called liquid hybridization capture-based bisulfite sequencing (LHC-BS) (Wang et al., 2011).

**TABLE 5:** Statistic of datasets in experiment.

	Liver Cancer Tissue	Normal Tissue	Total	Assay
GSE70090 (Li et al., 2016)	3	3	6	WGBS
GSE112221 (Hlady et al., 2019)	4	6	10	RRBS
GSE55752 (Gao et al., 2015)	8	8	16	Targeted BS
TCGA (Cancer Genome Atlas Research Network et al., 2013)	209	41	250	450K

The methylation levels captured on CpG locus were assigned to gene references based on homo sapiens (human) genome assembly GRCh37 (hg19). As multiple CpG locus were mapped to the same gene, we took the average of methylation levels across the whole gene region to represent the gene methylation score. For every data set, differential methylated genes were extracted using fold change, which is the absolute value of the difference between the means of tumor and non-tumor samples. We further analyzed the differential methylated genes from three data sets using three different assays and filtered out the commonly methylated genes. Moreover, we compared the functional enrichment of the commonly methylated genes with gene ontology (GO) (Ashburner et al., 2000; Gene Ontology Consortium, 2021), the pathways and connections of differential methylated gene from three data sets using Kyoto Encyclopedia of Genes and Genomes (KEGG) (Kanehisa and Goto, 2000).

Besides the three bisulfite sequencing methods, we also included Infinium Methylation 450 k Beadchip data from The Cancer Genome Atlas (TCGA) (Cancer Genome Atlas Research Network et al., 2013) for comparison. The differential methylated genes filtering and functional analysis followed the same procedure as bisulfite sequencing data.

## Results

### Procedures for Analyzing Bisulfite Sequencing Data

**FIGURE 1:**
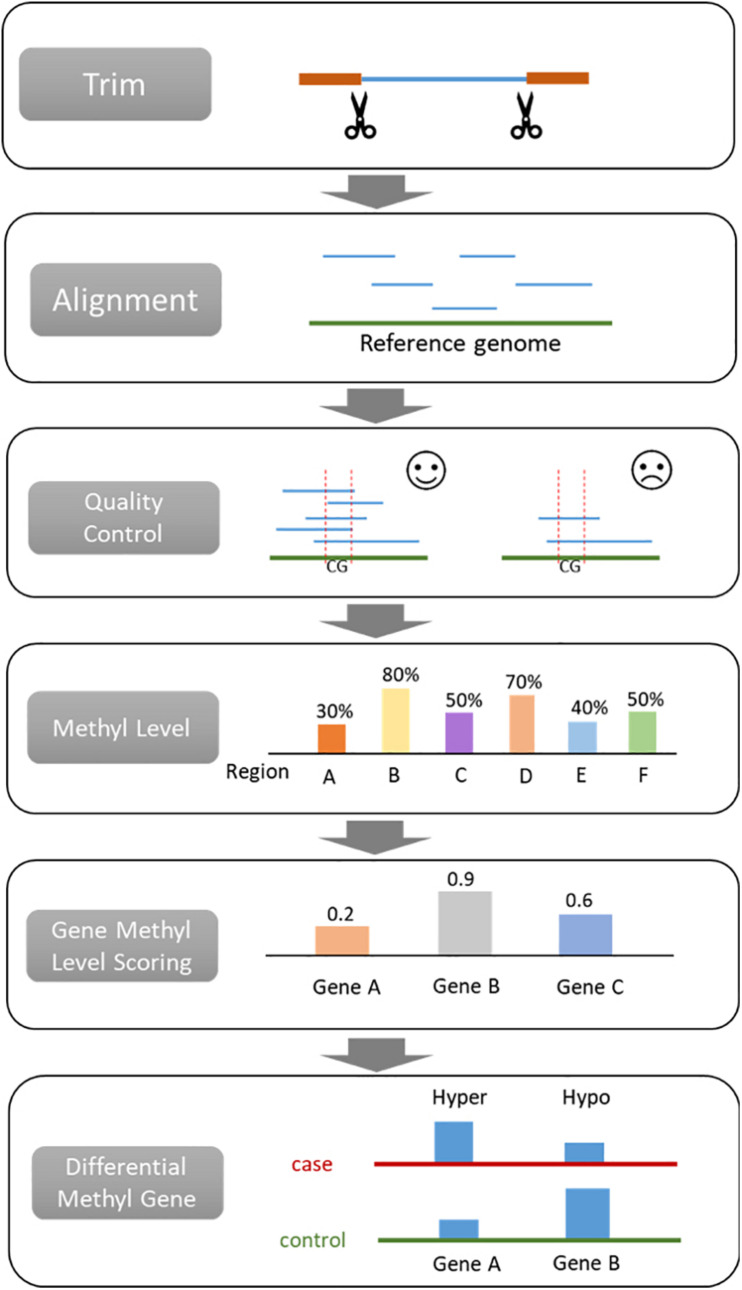
Procedures for analyzing bisulfite sequencing data.

A general bisulfite sequencing data process includes adapter trimming, alignment, quality control, methylation level scoring, and differential methylation region identification (Figure 1). Trimming aims to remove the sequence of adapters from reads, which are known sequences. The most widely used trimming method is Trim Galore! for WGBS, RRBS and targeted BS (Babraham, 2012). Most of the commercial DNA methylation assay kits provide the trimming tool. Generally, aligners also perform trimming because it affects the alignment result. This procedure is relatively fixed compared with other steps, resulting in only a few studies focusing on trimming. Hence, we do not discuss it in detail.

The bisulfite converted sequences require a specific aligner to map the reads to the reference genome. Quality control is applied for evaluating the quality of the assay and the alignment. By comparing the bisulfite sequence and the reference genome, the methylation level of loci or region can be calculated. Then differentially methylated regions (DMR) will be identified with statistical analysis according to the methylation level score. The normalization step is the same as gene expression (Cheng et al., 2016a,b; Liu et al., 2019) and hence not discussed in this paper. After that, researchers can perform downstream analysis depending on their research purposes, such as building machine learning classifiers for diagnosis or prognosis (Liu et al., 2020a,c; Wang et al., 2020a,b).

WGBS, RRBS and targeted BS have similar data format. Since RRBS focuses on a small partion of genomes, the read coverage of RRBS is higher than WGBS. Targeted BS has an even higher density of reads.

### Differential Methylated Genes Between WGBS, RRBS, Targeted BS and 450 k Microarray

By WGBS, RRBS, targeted BS and 450 k microarray, the methylated levels of chromosome location in GSE70090, GSE112221, GSE55752, and TCGA were measured. We mapped methylation levels to genes and summarized as gene methylated levels. 4,071, 15,059, 29,326, and 3,745 genes’ methylation levels were obtained from the four datasets, respectively, and 3,139 genes were shared.

**FIGURE 2:**
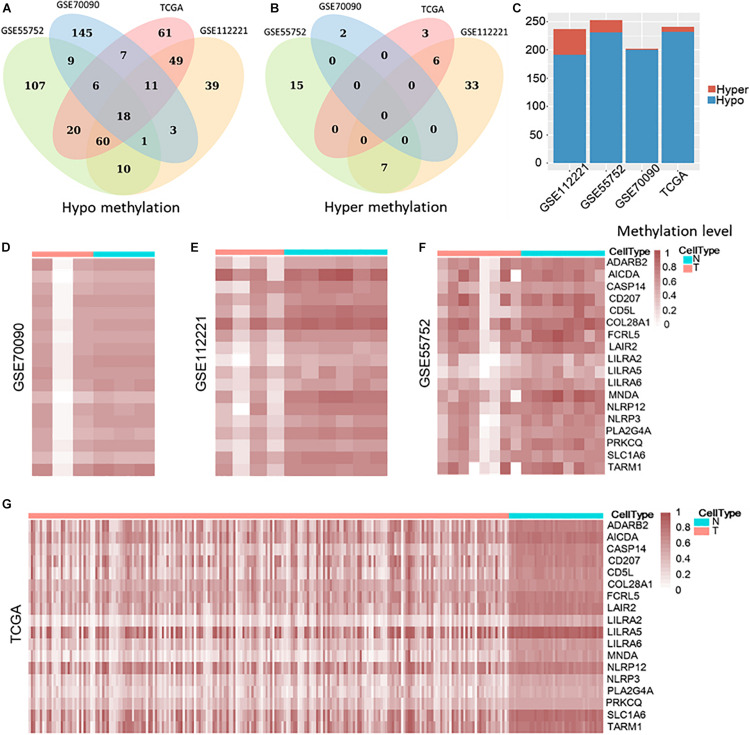
Hypo and hyper methylated genes in GSE70090, GSE112221, GSE55752, and TCGA **(A–C)**. Heat map of 18 common hypo-methylated genes in four datasets **(D–G)**.

Then, we compared the tumor and non-tumor samples within each dataset from the common genes and identified 202, 237, 253, and 241 differential methylated genes (DMG) in GSE70090 (WGBS), GSE112221 (RRBS), GSE55752 (targeted BS) and TCGA (450 k), respectively, with the threshold of fold change > 0.15. Our result illustrated that most differentially methylated genes (DMGs) were hypo methylation (Figures 2A,C) while few were hyper methylation (Figures 2B,C). Specifically, 200, 191, 231, and 232 genes were hypo-methylated in GSE70090, GSE112221, GSE55752, and TCGA (Figure 2A). Nine differential genes were exclusively shared among the three bisulfite sequencing datasets and 18 genes were common across the four datasets.

The 18 differential methylated genes were ADARB2, AICDA, CASP14, CD207, CD5L, COL28A1, FCRL5, LAIR2, LILRA2, LILRA5, LILRA6, MNDA, NLRP12, NLRP3, PLA2G4A, PRKCQ, SLC1A6, and TARM1. These common differential genes were all hypo-methylated in the four liver cancer datasets (Figures 2D–G).

### Functional Analysis of 18 Common Differential Methylated Genes

We explored the topological properties of the 18 common DMGs, including degree, betweenness and transitivity, which have been widely used in cancer and disease analysis (Cheng and Leung, 2018b; Cheng et al., 2019; Liu et al., 2020b). In the protein-protein interaction (PPI) network, TARM1, PRKCQ and MNDA are the top three genes with the highest network connectivity. PLA2G4A, PRKCQ, CD207, and MNDA have high betweenness, which measures the extent to which a gene lies on the shortest paths between other genes. The transitivity of COL28A1, CASP14, TARM1 and MNDA is over 0.2, indicating their interactors are prone to cluster together. Notably, Myeloid Cell Nuclear Differentiation Antigen (MNDA) is at a high level in all the three topological metrics, suggesting it is an important transcriptional regulator in liver cancer.

**FIGURE 3:**
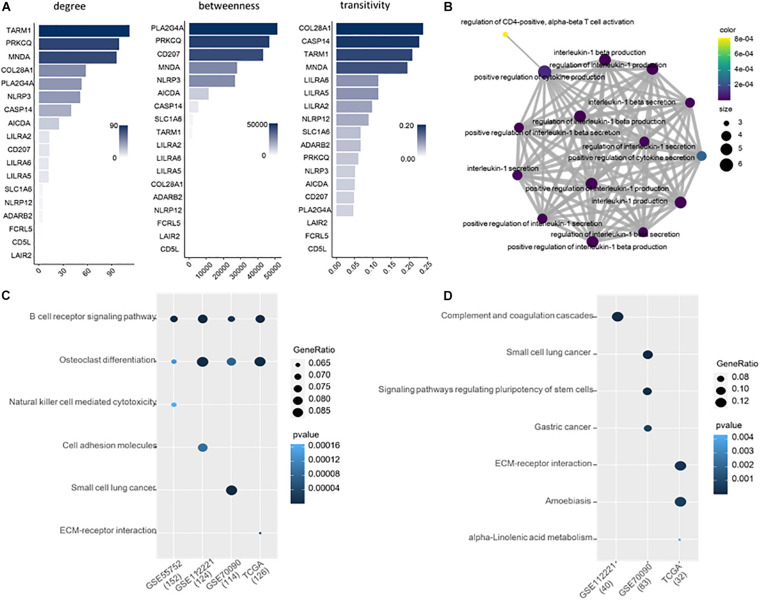
**(A)** Ranking of the 18 DMGs by topological importance. **(B)** Enrichment analysis of the 18 genes. **(C)** Enriched KEGG pathways for the DMGs of each of the four datasets. **(D)** Enriched KEGG pathways for the DMGs exclusive in each dataset.

We further analyzed the 18 common DMGs using Gene Ontology (GO) (Gene Ontology Consortium, 2021). They are significantly involved in the 15 immune-related functions (Figure 3). 12 out of 15 belong to the regulation of interleukin-1, i.e., interleukin-1 production, interleukin-1 secretion, interleukin-1 beta production, interleukin-1 beta secretion, positive regulation of interleukin-1 beta secretion, regulation of interleukin-1 beta production, etc. Interleukin-1 is a family of cytokines related to liver diseases (Tsutsui et al., 2015; Barbier et al., 2019). They also overrepresented in three other functions, positive regulation of cytokine production, positive regulation of cytokine secretion and regulation of CD4-positive, and alpha-beta T cell activation, all of which are of importance in cancer development and progression.

### Pathways of Differential Methylated Genes in Four Datasets

We applied Kyoto Encyclopedia of Genes and Genomes (KEGG) to study the biological pathways that the DMGs involved. 152, 124, 114, and 126 genes from GSE55752, GSE112221, GSE70090, and TCGA were included in the KEGG database. The methylated genes from the four datasets are consistently involved in B cell receptor signaling pathway and osteoclast differentiation (Figure 3C).

Moreover, we executed pathway enrichment analysis using the DMGs exclusively identified from the four datasets. 40, 83 and 32 genes were detected from GSE112221, GSE70090, and TCGA, respectively, which are enriched in the pathways of complement and coagulation cascades, small cell lung cancer, amoebiasis, etc. (Figure 3D). In comparison to the DMGs of each dataset, the exclusive ones are implemented in distinct pathways that are functionally inconsistent.

We also enriched the DMGs in functional categories of GO (Supplementary Figures 1, 2). Similarly, the results illustrated that the genes found in the four datasets have some terms in common.

### Functional Correlation Between WGBS, RRBS, Targeted BS, and TCGA

**FIGURE 4:**
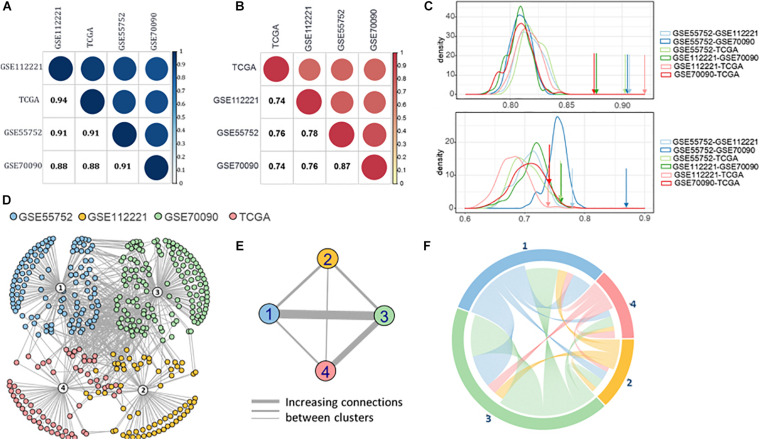
**(A)** Semantic similarity of all the differential methylated genes from four datasets. **(B)** Semantic similarity of the differential methylated genes unique in four datasets. **(C)** Density of semantic similarity of two genes randomly picked up from two datasets. **(D)** Protein-protein association network of four datasets. **(E)** Connection across the four datasets. **(F)** Functional association between four datasets.

Based on the semantic similarity of GO terms, we calculated the functional similarity of the DMGs across the four datasets. These DMGs are highly consistent with all the semantic similarity scores higher than 0.88 (Figure 4A). Importantly, the dataset-exclusive DMGs also obtain a high score (>0.74, Figure 4B), which is significantly higher than that of the simulated genes (Figure 4C).

Moreover, we retrieved the proteins regulated by the DMGs to compute the protein-protein association (Cheng et al., 2017; Cheng and Leung, 2018a; Li et al., 2020) and formed the protein-protein interaction (PPI) network using STRING v11 (Szklarczyk et al., 2019; Figure 4D). A high proportion of the methylated genes of the four datasets are closely interacted, revealing a high connection between methylated genes in the four methylation measuring methods. Furthermore, GSE55752 (blue) and GSE70090 (green) are connected most closely in the network followed by the connection between GSE70090 (green) and TCGA (red; Figure 4E). The connections were reorganized in Figure 4F and GSE70090 shows a strong correlation with other datasets.

## Discussion

This study introduced three types of bisulfite sequencing measurements for DNA methylation and compared different approaches for aligning bisulfite-converted reads, assessing quality, calculating methylation level, and calling differentially methylated regions. Datasets of liver cancer measured by WGBS, RRBS, and targeted BS were preprocessed and DMGs were screened. We observed that the common DMGs across different technologies are consistently hypo-methylated, which is consistent with our previous discoveries that genes tend to up-regulated in cancers (Cheng et al., 2016a,b; Liu et al., 2019). We further compared the functional enrichment analysis of the three datasets and found the DEMs of the three assays are functionally and semantically similar.

We compared the accuracy, efficiency, and mapping ability of the aligners according to the experimental results reported in other papers. For accuracy, the ranking is as follows, BathMeth2, VAliBS, BS-Seeker3 > BSMAP > Bismark, BiSpark > Brat-Nova. As for efficiency, the ranking is BiSaprk > BS-Seeker3 > BSMAP > Brat-Nova > Bismark > VAliBS. For map ability, the preference is BatMeth2, VAliBS > BiSpark, BS-Seeker3,Brat-Nova > BSMAP > Bismark. Some aligners are not implemented and compared by other papers, so we listed them in the same rank.

The choice of aligners depends on not only the research objectives but also the data and researchers’ situation. For a small amount of data, BatMeth2 (Zhou et al., 2019) is recommended because of its accuracy and map ability. On the other hand, BiSpark (Soe et al., 2018) is better for a large amount of data. For researchers not good at programming, ViAliBS (Li et al., 2017) is more user-friendly for its graphical user interface.

The features of quality control methods are summarized in Table 2. The cloud-based platform provides the most comprehensive metrics for quality control, while MethGo has better visualization of the results. GBSA can also be adopted for non-programmers.

As for methylation level scoring, Table 3 shows that BSPAT (Hu et al., 2015) outperforms other tools for its significance of scoring, visualization of results, capability for large data, and user friendliness.

For DMR identification, it depends on the research topics for the choice of methods. For the pairwise situation such as methylation in health and disease, DMRFusion can be chosen because it makes use of different types of models. If the spatial information is important for the study, MethCP may be a better choice as well as for time course problem.

When using bisulfite sequencing methods to detect methylation, WGBS has the highest coverage and resolution followed by RRBS, but targeted BS is cost-effective. The microarray technique (450 K in this paper) has the lowest coverage and resolution compared with bisulfite sequencing. Although WGBS, RRBS, and targeted BS have different coverage of CpG locus, the commonly detected DMGs have high similarity in functions and the common genes are consistently hypo-methylated in liver cancer. Besides, 450 K is also comparable in detecting DMGs in liver cancer without considering its low resolution.

## Data Availability Statement

The datasets presented in this study can be found in online repositories. The names of the repository/repositories and accession number(s) can be found in the article/Supplementary Material.

## Author Contributions

LC and XZ conceived of the presented idea. XZ and QW prepared the data and performed the computations. HW and LC analyzed the results. XL, KL, and MW supervised this work. LC and XZ wrote and revised the manuscript. All authors contributed to the article and approved the submitted version.

## Conflict of Interest

The authors declare that the research was conducted in the absence of any commercial or financial relationships that could be construed as a potential conflict of interest.
