# Supplementary material for: Evaluating the Consistency of Gene Methylation in Liver Cancer Using Bisulfite Sequencing Data
Source: Front Cell Dev Biol. 2021 Apr 29;9:671302. doi: 10.3389/fcell.2021.671302 (PMC8116545; doi:10.3389/fcell.2021.671302)
Supplement: Supplementary file 1 [file Data_Sheet_1.DOCX]

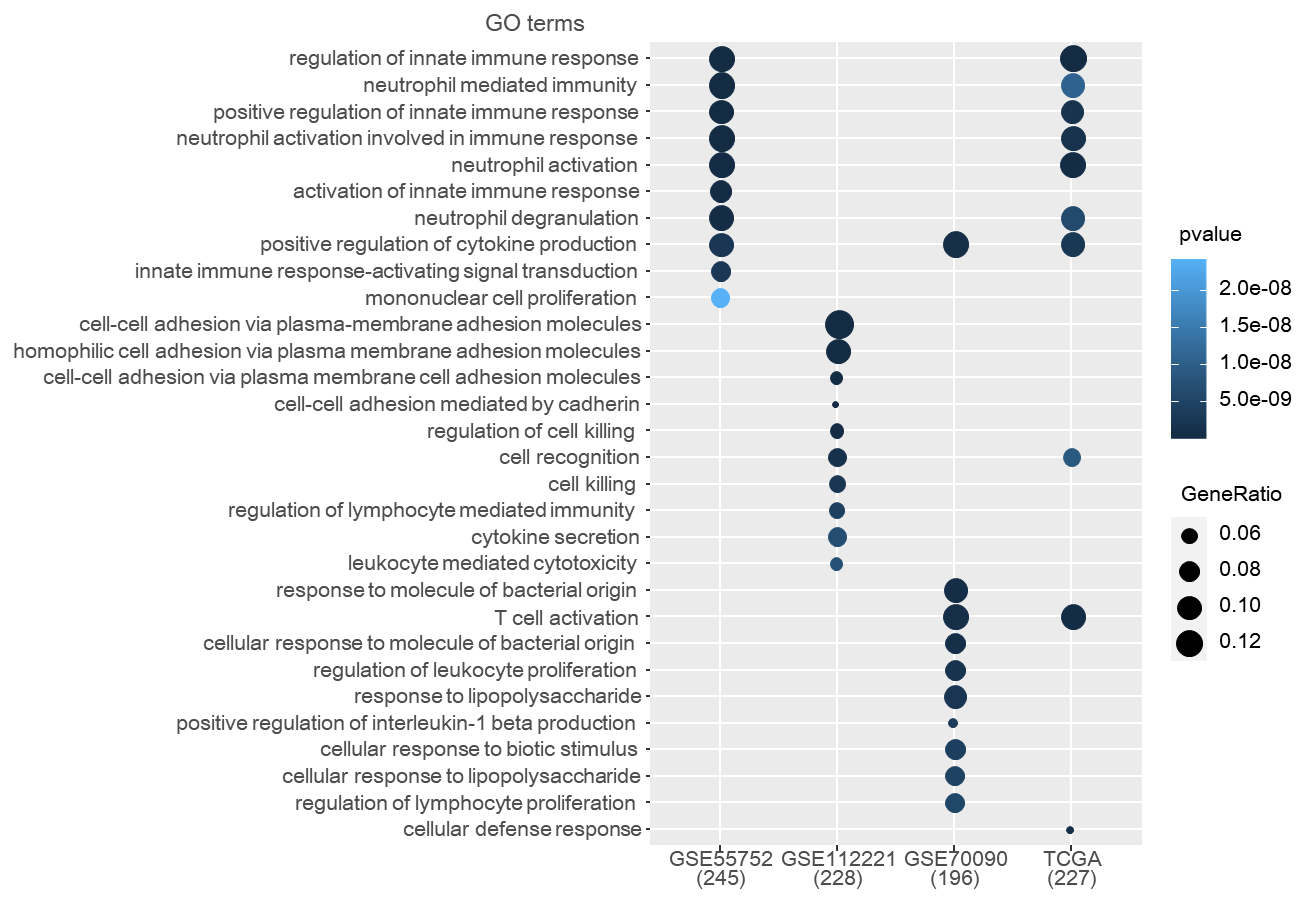


**Figure** S1. All differential methylated genes in the four datasets enriched in gene ontology (GO) terms.


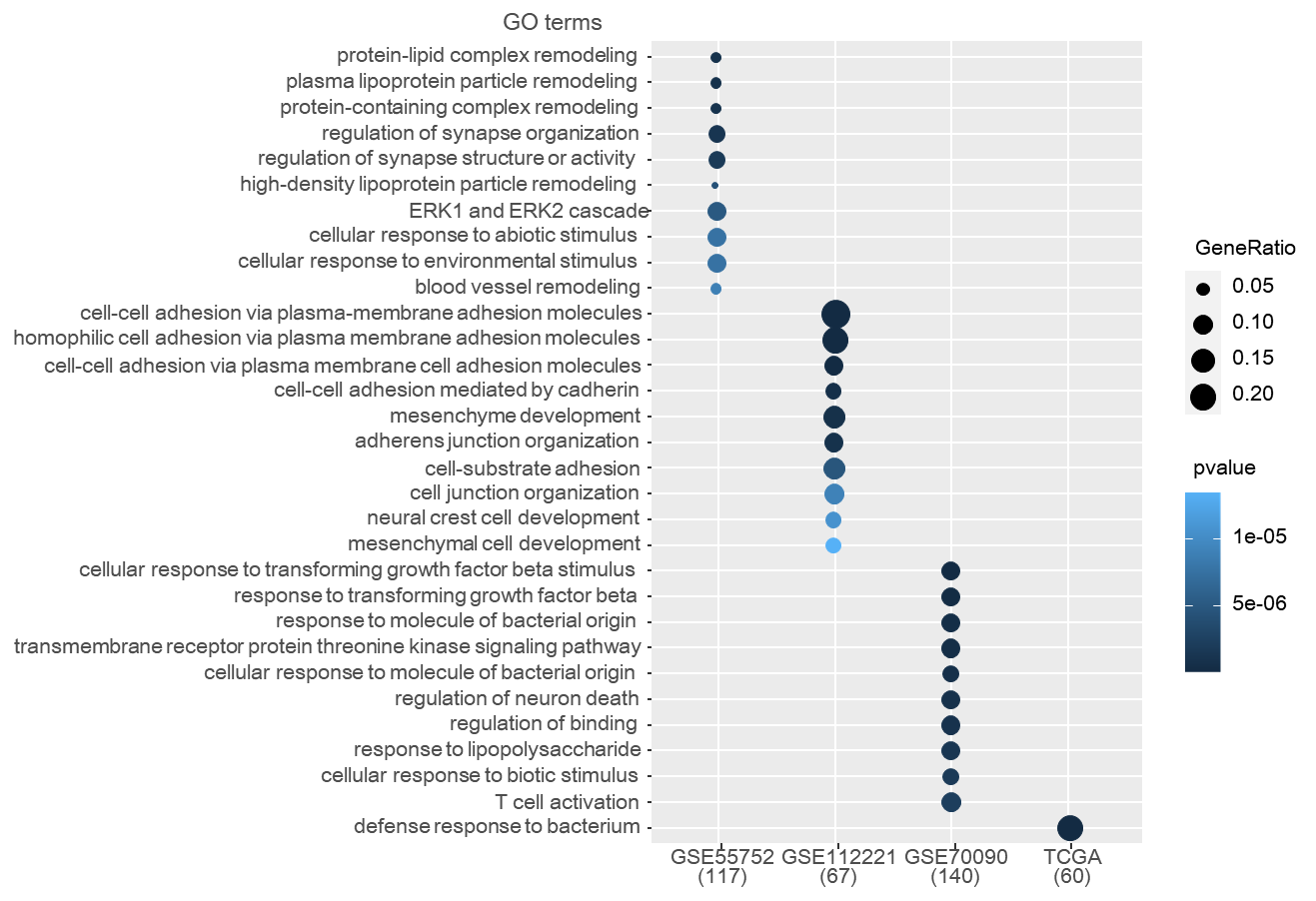


**Figure** S2. Exclusive differential methylated genes in the four datasets enriched in gene ontology (GO) terms.
